# Morgagni−Larrey diaphragmatic hernia repair in adult patients: a retrospective single-center experience

**DOI:** 10.1007/s10029-020-02147-0

**Published:** 2020-02-29

**Authors:** P. U. Oppelt, I. Askevold, F. Bender, J. Liese, W. Padberg, A. Hecker, M. Reichert

**Affiliations:** Department of General, Visceral, Thoracic, Transplant and Pediatric Surgery, University Hospital of Giessen, Giessen, Germany

**Keywords:** Congenital diaphragmatic hernia, Morgagni hernia, Larrey hernia, Mesh reinforcement, Primary suture

## Abstract

**Purpose:**

Morgagni−Larrey congenital diaphragmatic hernia (MLH) is rare in adult patients and surgery is performed infrequently. The evidence regarding the most beneficial treatment modality is low. Nevertheless, with increasing experience in minimally-invasive surgery, the literature proves the laparoscopic approach as being safely feasible. However, knowledge on the disease as well as treatment options are based on single surgeon’s experiences and small case series in the literature.

**Methods:**

Retrospective single-center analysis on adult patients (≥ 18 years) with MLH from 01/2003 to 06/2019 regarding symptoms, hernia sac contents, surgical technique and perioperative outcome.

**Results:**

4.0% of diaphragmatic hernia repair procedures were performed for MLH (*n* = 11 patients). 27.3% of these patients were asymptomatic. Dyspnea or gastrointestinal symptoms were frequently observed (both in 45.5% of the patients). Colon transversum (63.6%), omentum majus (45.5%) and/or stomach (27.3%) were the most common hernia sac contents. Correct diagnosis was achieved preoperatively in 10/11 patients by cross-sectional imaging. All procedures were performed by trans-abdominal surgery (laparotomy in four and laparoscopy in seven patients). All hernias were reinforced by mesh after primary closure. No differences were observed in the perioperative outcome between patients who underwent hernia repair by laparotomy versus laparoscopy. Pleural complications requiring drainage were the most common postoperative complications.

**Conclusion:**

MLH repair seems to be safely feasible by laparoscopic surgery. The benefit of mesh augmentation in MLH repair is not clear yet. In contrast to the current literature, all patients in this study received mesh augmentation after primary closure of the hernia. This should be evaluated in larger patient cohorts with long-term follow-up.

## Introduction

Morgagni−Larrey hernias (MLH) are the most common congenital defects in the anterior, parasternal portion of the diaphragm. The incidence of these non-traumatic retrocostoxiphoid hernias is estimated to be approximately 1–5% of all types of congenital diaphragmatic hernias and remains even more uncommon in adult patients [1–3]. Although MLH is most frequently diagnosed in neonates and children, the hernia can remain undiagnosed until adulthood, due to unspecific symptoms or if the patients are not compromised [1, 4]. Nevertheless, diagnosis and treatment of MLH in adult patients are relatively uncommon and rare. In the current literature, the frequency of right-sided (Morgagni) retrocostoxiphoid hernias in children is the highest with approximately 90%, followed by bilateral MLH (8%) and left-sided (Larrey) retrocostoxiphoid hernias (2%), respectively [2, 3, 5]. Thereby, the exact prevalence of MLH in adult patients remains unclear. If diagnosis of MLH was not made incidentally in the asymptomatic adult patient (approximately 30% of the cases), symptoms of the patients are unspecific and range from dyspnea to abdominal or chest pain and constipation in approximately 36%, 37% and 20% of the cases, respectively [1, 4, 6]. Caused by the rarity of MLH in adult patients, the knowledge about symptoms, adequate diagnosis, imaging techniques and appropriate treatment modalities is only based on single surgeon’s experiences as well as case reports and small case series in the current literature. Neither prospectively conducted trials or larger retrospective studies with a long-term follow-up of the patients after MLH repair are available from the current literature, nor recommendations for surgical treatment of adult patients with MLH are obtained from respective medical societies. Following the literature review, questions may arise for the treating surgeon regarding the appropriate surgical approach, closure technique of the hernia or—in accordance with the hiatal hernia repair—if a mesh augmentation should be performed after primary suture of the hernial orifice. However, it seems to be important to improve the evidence by providing single institutional experiences along with our patient cohort and a technical section with a *how-I-do-it* proposal for the treatment of anterior retrocostoxiphoid MLH.

## Materials and methods

### Patients and study design

We retrospectively evaluated adult patients who underwent diaphragmatic hernia repair from 01/2003 to 06/2019 at the University Hospital of Giessen. The retrospective data acquisition was formally approved by the local ethics committee of the medical faculty of the University of Giessen (AZ 97/19). Each patient was treated by the local standard-of-care.

To exclusively focus on congenital MLH, patients who underwent isolated hiatal hernia repair, posterior, lumbocostal diaphragmatic (Bochdalek) hernia repair as well as posttraumatic hernia repair with trauma being the underlying cause for hernia development were excluded primarily from the analysis. Infant patients who suffered from congenital diaphragmatic defects and patients who were operated on under the age of 18 were also excluded from the study to solely focus on the rare condition of congenital anterior diaphragmatic MLH repair in adult patients.

Mean outcome parameters we focused on were symptoms of the patients, indication for surgery, the surgical technique, use of mesh devices for augmentation, operation time and postoperative stay on intensive care unit (ICU) as well as total postoperative in-hospital stay. Patient data and patient characteristics were evaluated retrospectively from the prospectively maintained institutional database.

After a primary analysis of patient data we divided the patients into the group who underwent minimally-invasive, laparoscopic surgery (group_LSK) and the group who underwent primary open hernia repair by laparotomy (group_LAP) to compare the feasibility and safety of a minimally-invasive surgical approach for the MLH. Statistical analysis was performed using GraphPad Prism (Version 5.00 for Windows, GraphPad Software, San Diego California USA, www.graphpad.com) for two group comparisons. Data of both groups were analyzed using Fisher’s exact or Pearson’s X^2^ test for categorical data in cross-tabulation. Two-group comparisons were performed by Mann–Whitney−U test. Data are given as median and range; *p* values ≤ 0.05 were considered to indicate statistical significance.

### Surgical technique

Patients who underwent open surgical diaphragmatic hernia repair were placed in supine position and a median laparotomy was used to access the epigastric region under general anesthesia with single-lumen intubation. Hernia sac contents were gently reduced and removed to the abdominal cavity as the first step of hernia repair. Hernia sacs were excised and resected as the standard in all patients. The diaphragmatic defect was closed primarily with thick, non-resorbable, interrupted sutures. We used mesh reinforcement after the primary suture hernia repair in all patients reported in this retrospective study (although, in hiatal hernia repair we use mesh augmentation depending on the size of the hernia). The size of the mesh was considered to overlap the repair site approximately at 3−5 cm beyond the edge of the defect. Single sutures or endo-staplers are used for fixation of the mesh.

In laparoscopic, minimally-invasive surgery for MLH repair we basically follow the same principles as in open surgery. Here, patients are placed in reverse Trendelenburg position. The mesh is fixed in laparoscopic surgery using endo-stapler devices. The institutional operation technique for laparoscopic MLH repair with mesh augmentation is adopted from the clinical standard in hiatal hernia repair.

### Literature review

To improve the evidence and the discussion of manuscript data, a literature review on congenital, non-traumatic, diaphragmatic hernia repair with focus on adult patients (MLH and Bochdalek diaphragmatic hernia) was performed. Medline was searched on July, 2019, using the terms “Larrey OR Morgagni OR Bochdalek”, “congenital diaphragmatic hernia AND adult”, “congenital diaphragmatic hernia AND elderly”, “congenital diaphragmatic hernia repair”. Case series published from 01/2005 to 06/2019 containing ≥ 5 adult cases treated for congenital, non-traumatic, diaphragmatic hernia were included in the literature review.

## Results

272 surgical procedures on any type of diaphragmatic hernia were performed in 252 patients during the study period at our institution, including 20 re-do surgeries for any type of diaphragmatic hernia: 217 patients underwent 234 surgical procedures for hiatal hernia repair, 2 patients underwent posttraumatic diaphragmatic hernia repair, 1 adult patient underwent Bochdalek posterior diaphragmatic hernia repair and 21 infant patients (up to the age of 18 years) underwent 24 surgical procedures for congenital hernia repair, respectively.

11 adult patients—4.0% of all surgical procedures performed for diaphragmatic hernia during this 17.5- year observational period—underwent anterior diaphragmatic MLH repair and were included into this retrospective single-center analysis.

Patient characteristics with regard to the side of the hernia (left-sided versus right-sided) as well as to the surgical approach (primary laparoscopic versus primary open surgery) are given in Table 1. In comparison between the groups of patients who underwent primary open or laparoscopic surgery, patient characteristics and comorbidities were widely balanced.

**Table 1:** Patient characteristics

Variables	All patients (*n* = 11)	Subgroup analysis					
		Hernia specific			Procedure specific		
		Right-sided Morgagni hernia (*n* = 4)	Left-sided Morgagni hernia (*n* = 7)	*p* value	Open surgery (*n* = 4)	Laparoscopic surgery (*n* = 7)	*p* value
Male gender	10 (90.9%)	4 (100%)	6 (85.7%)	1	4 (100%)	6 (85.7%)	1
Age (years)	53 (19–68)	50 (37–68)	53 (19–66)	0.85	43 (37–68)	60 (19–66)	0.93
BMI (kg/m2)	28.7 (20.1–37.0)	32.1 (27.7–37.0)	24.7 (20.1–32.7)	0.11	31.1 (27.7–34.7)	24.7 (20.1–37.0)	0.23
Open surgery Laparoscopic surgery	− −	3 (75%) 1 (25%)	1 (14.3%) 6 (85.7%)	− −	− −	− −	− −
Chronic diseases Cardiac Pulmonal Renal	9 (81.8%) 7 (63.6) 4 (36.4%) 2 (18.2%)	4 (100%) 3 (75%) 1 (25%) 1(25%)	5 (71.4%) 4 (57.1%) 3 (42.9%) 1 (14.3%)	0.49 1 1 1	4 (100%) 3 (75%) 1 (25%) 0	5 (71.4%) 4 (57.1%) 3 (42.9%) 2 (28.6%)	0.49 1 1 0.49
Symptoms							
None Dyspnea Gastrointestinal^a^ Postprandial abdominal discomfort	3 (27.3%) 5 (45.5%) 5 (45.5%) 3 (27.3%)	0 3 (75%) 1 (25%) 0	3 (42.9%) 2 (28.6%) 4 (57.1%) 3 (42.9%)	0.24 0.24 0.55 0.24	0 3 (75%) 2 (50%) 3 (75%)	3 (42.9%) 2 (28.6%) 3 (42.9%) 3 (42.9%)	0.24 0.24 1 0.55
Contents of the hernia							
Stomach Colon transversum Small intestine Liver Spleen Omentum majus	3 (27.3%) 7 (63.6%) 2 (18.2%) 1 (9.1%) 1 (9.1%) 5 (45.5%)	0 3 (75%) 0 1 (25%) 0 3 (75%)	3 (42.9%) 4 (51.1%) 2 (28.6%) 0 1 (14.3%) 2 (28.6%)	0.24 1 0.49 0.36 1 0.24	1 (25%) 3 (75%) 0 1 (25%) 0 1 (25%)	2 (28.6%) 4 (51.1%) 2 (28.6%) 0 1 (14.3%) 4 (51.1%)	1 1 0.49 0.36 1 0.55

*BMI*, Body mass index

^a^Including reflux disease

As shown in Table 1, most patients suffered from dyspnea (*n* = 5; 45.5%) or gastrointestinal symptoms (*n* = 5; 45.5%, most commonly postprandial abdominal discomfort). In three patients (27.3%) diagnosis was made incidentally. Diagnosis of the MLH was made preoperatively in ten patients, all of those patients received computed tomography (CT) or magnetic resonance tomography preoperatively. In one patient the MLH was found and treated minimal-invasively during laparoscopic hiatal hernia repair. In the aforementioned patient a barium swallow X-ray examination was not sufficient to preoperatively diagnose the MLH correctly. One patient in the group_LSK suffered from recurrence of left-sided MLH in the long-term follow-up after congenital hernia repair in the childhood and was consecutively treated by laparoscopic re-do hernia repair as an adult at this time.

In the patient collective, MLH repair was performed four times by primary open abdominal surgery with a median laparotomy and seven times primarily by minimally-invasive laparoscopic surgery. No conversions from the initially intended laparoscopic approach to open surgery were observed during the study period. One patient with a right-sided MLH in the group_LAP suffered from incarceration of the liver and underwent an open thoraco-abdominal approach in the emergency setting with mesh augmentation from both sides of the diaphragm. The most common content of the hernia sac was colon transversum (*n* = 7; 63.6%), omentum majus (*n* = 5; 45.5%) and/or stomach (*n* = 3; 27.3%). In all patients the MLH was primarily sutured and finally reinforced with a mesh. During the study period different mesh implants were used following the current institutional standard-of-care in ventral abdominal or hiatal hernia repair, including coated mesh devices (*n* = 6), uncoated mesh devices (*n* = 4) and one Gore-Tex^®^ mesh device. Table 2 provides further information regarding the size of mesh implants. Three patients from the group_LAP received a thoracic drain intraoperatively versus none from the group_LSK (*p* = 0.02). The total duration of the surgical procedure was 106.5 (97–229) minutes in the group_LAP and 120 (90–201) minutes in the group_LSK (*p* = 0.79). Postoperative stay on ICU as well as the total postoperative in-hospital stay was 0 (0–6) days in the group_LSK and 2 (1–3) days in group_LAP as well as 6 (3–23) days in group_LSK and 8 (7–16) days in group_LAP, respectively, with no statistically significant differences between both groups (Table 2).

**Table 2:** Perioperative outcome

Variables	All patients (*n* = 11)	Subgroup analysis					
		Hernia specific			Procedure specific		
		Right-sided Morgagni hernia (*n* = 4)	Left-sided Morgagni hernia (*n* = 7)	*p* value	Open surgery (*n* = 4)	Laparoscopic surgery (*n* = 7)	*p* value
Operation duration (min)	109 (90–229)	101.5 (90–229)	120 (105–201)	0.32	106.5 (97–229)	120 (90–201)	0.79
Mesh size (cm2)^a^	300 (66–1050)	300 (150–600)	300 (66–1050)	1	600 (300–1050)	225 (66–560)	0.0878
Postoperative in-hospital stay (days)							
ICU Total	1 (0–6) 7 (3–23)	2 (0–3) 7.5 (4–16)	1 (0–6) 7 (3–23)	0.49 0.85	2 (1–3) 8 (7–16)	0 (0–6) 6 (3–23)	0.15 0.25
Postoperative complications							
*n* patients *n* complications	6 (54.5%) 9	2 (50%) 3	4 (57.1%) 6	1 −	2 (50%) 3	4 (57.1%) 6	1 −
Postoperative complications^b^ [median (range)]	3 (1–3)^c^	2 (1–3)	3 (1–3)	−	2 (1–3)	3 (1–3)	−
Grade I Grade II Grade IIIa	3 1 4	2 0 1^d^	1 1 3^e^	−	2 0 1^d^	1 1 3^e^	−

*ICU*, Intensive care unit

^a^Data on mesh size were not available retrospectively in two patients (i.e. one patient from both the open and laparoscopic surgery group or the right-sided and left-sided hernia group, respectively)

^b^Complications during the initial hospital stay regarding the Clavien-Dindo classification of surgical complications [7]. One patient with a left-sided hernia in the laparoscopic surgery group was readmitted to hospital due to recurrent pleural effusion requiring pleural re-drainage

^c^Including one patient with a pleural effusion not requiring drainage

^d^Pleural effusion requiring drainage

^e^Including pleural effusion in two patients and hemothorax in one patient requiring drainage

Reflecting a lower extent of tissue trauma during surgery, early postoperative C-reactive protein (CRP) and blood leucocyte values were slightly different between both groups, showing higher values in patients after open abdominal surgery compared to laparoscopic surgery (Table 3). Nevertheless, no differences were observed in the rate of postoperative complications (stratified by the Clavien–Dindo classification of surgical complications [7] in Table 2). Overall, patients suffered most frequently from pleural complications during the postoperative hospital stay. Four patients developed serothorax postoperatively, and three of them required drainage. One patient suffered from postoperative hemothorax and required thoracic drainage (Table 2). One patient from the group_LSK was readmitted to the hospital due to a recurrent pleural effusion which was treated repeatedly by thoracic drainage 30 days after the operation.

**Table 3:** Perioperative inflammatory markers

Variables	All patients (*n* = 11)	Subgroup analysis					
		Hernia specific			Procedure specific		
		Right-sided Morgagni hernia (*n* = 4)	Left-sided Morgagni hernia (*n* = 7)	*p *value	Open surgery (*n* = 4)	Laparoscopic surgery (*n* = 7)	*p* value
Perioperative leucocytes (giga/l)							
Preoperative POD 1 POD 2 POD 3 POD 4	7.6 (4.8–9.8) 11.6 (5.9–22.6) 10.0 (5.1–17.3) 8.5 (4.1–13.9) 9.1 (6.4–16.0)	9.3 (8.1–9.8) 12.9 (9.3–22.6) 15.1 (10.0–17.3) 10.2 (9.7–13.9) 9.7 (9.2–16.0)	5.8 (4.8–8.2) 7.9 (5.9–14.3) 8.2 (5.1–11.1) 7.4 (4.1–9.4) 8.6 (6.4–10.3)	0.01 0.16 0.02 0.006 0.14	8.7 (8.1–9.8) 14.1 (11.9–22.6) 15.1 (10.7–17.3) 10.0 (8.5–13.9) 9.5 (8.6–16.0)	5.8 (4.8–9.4) 7.9 (5.9–13.1) 8.2 (5.1–11.1) 7.4 (4.1–10.0) 7.8 (6.4–10.3)	0.04 0.01 0.01 0.04 0.34
Perioperative CRP (mg/l)							
Preoperative POD 1 POD 2 POD 3 POD 4	2.0 (0.5–18.6) 39.8 (0.6–202.1) 90.6 (20.5–206.3) 93.3 (0.6–214.2) 97.1 (1.0–145.4)	7.8 (1.2–18.6) 48.5 (37.2–55.1) 125.8 (47.7–206.3) 136.0 (51.6–214.2) 126.2 (114.7–145.4)	1.0 (0.5–10.3) 38.8 (0.6–202.1) 90.6 (20.5–154.1) 88.3 (0.6–151.4) 70.1 (1.0–122.7)	0.11 0.53 0.53 0.16 0.07	5.2 (1.2–18.6) 51.9 (48.2–69.8) 152.2 (90.2–206.3) 136.0 (25.7–214.2) 120.4 (1.0–145.4)	1.0 (0.5–10.3) 37.2 (0.6–202.1) 80.7 (20.0–154.1) 88.3 (0.6–151.4) 74.8 (35.8–122.7)	0.23 0.07 0.11 0.23 0.49

*CRP* C-reactive protein; *POD* postoperative day

None of the patients was readmitted to our hospital for recurrence of the MLH during a median follow-up observational period of 24 (7–165) months, i.e. the time from hernia repair until 06/2019.

## Discussion

The congenital defect of the diaphragm leading to MLH is most commonly a condition in pediatric patients [4]. Only a minority of diaphragmatic hernias in adults are congenitally originated MLHs, thus surgery for MLH is rarely performed even in high volume centers. The whole evidence regarding incidence, clinical symptoms, diagnosis and treatment is only based on single case reports and small retrospective case series as well as single surgeon´s or single institutional experiences. In their literature review Horton et al. summarized 298 adult patients suffering from MLH published in 135 articles during a period of 55 years [4]. The fact, that most of the articles report single cases, reflects and underlines the rarity of the disease in adults [4]. Even in this literature review approximately one third of the patients were asymptomatic. Otherwise, the symptoms were unspecifically similar to our study: abdominal or thoracic pain in 37% of the patients, pulmonary symptoms in 36% and obstruction in 20%. Contrastingly, dysphagia or chronic gastroesophageal reflux disease was quite rare [4]. Therefore, an association between hernia size and symptoms of the patients had been controversially discussed in the current literature [4]. Nevertheless, Sirmali et al. discussed small MLH being more often asymptomatic [8]. Young et al. recently reported the largest retrospectively conducted single-center analysis in the current literature [1]. Even herein, 19% of the patients were asymptomatic and 36% suffered from respiratory symptoms, 29% from gastrointestinal symptoms, and 17% from both [1]. Unfortunately, they did not differentiate the single gastrointestinal symptoms from the reflux disease, obstruction et cetera to make them comparable to other published case series or our study in more detail. Hence, Young et al. gave some information about the hernia sac contents with the large intestine and omentum being the most commonly prevalent sac contents in their patient cohort [1]. The stomach was affected by the MLH in a minority of the cases [1]. This is comparable to our study (see Table 1) and finally the hernia sac contents may explain the symptoms of the patients [1]. The accurate evaluation of symptoms of the affected patients is quite difficult retrospectively and constitutes a strong limitation of retrospectively conducted studies. However, the symptoms of the patients are surely dependent on hernia sac contents with the small or large intestine leading to obstruction, stomach leading to postprandial abdominal discomfort or reflux disease, and omentum probably leading to pain whereas it might be dependent on the hernia size, if the patients complain of pulmonary complications, dyspnea or exercise intolerance. Beneath clinical symptoms, appropriate imaging is mandatory for accurate diagnosis and planning of the adequate therapeutic concept. In our study 10 of 11 patients received any type of cross-sectional imaging (computed tomography or magnetic resonance tomography) in the preoperative phase, which led to the correct diagnosis preoperatively. This should be recommended in patients, in whom an extra-hiatal diaphragmatic hernia is suspected. One patient in our cohort received conventional imaging (barium swallow examination for coexistent hiatal hernia) for the diagnosis of hiatal hernia, which fails to accurately depict the MLH. In this case the MLH was found and synchronously treated during laparoscopy for the hiatal hernia. Also Testini et al. as well as Ohtsuka and Suzuki recommended a cross-sectional imaging technique, in particular CT scan as the diagnostic tool of choice for extra-hiatal diaphragmatic hernia [9, 10]. Cross-sectional imaging techniques are not only sufficient to precisely diagnose the presence, size and localization as well as the possible complications of the hernia like intestinal strangulation but also to exclude the whole bundle of differential diagnoses of extra-hiatal diaphragmatic hernia [4, 9, 10]. Due to a high rate of incidental finding of MLH especially in asymptomatic patients, the exact prevalence of MLH in adults is not known [1]. As recently discussed by Young et al. the improvement of cross-sectional imaging techniques and the higher frequency of cross-sectional imaging techniques in clinical application lead to higher rates of incidental diagnosis of congenital diaphragmatic hernia even in asymptomatic patients [1]. Beneath the minimal growth rate per year of the hernia which is making the repair more difficult and complicative, an early hernia repair should also prevent severe complications of the hernia like incarceration, volvulus and/or obstruction of the sac content even in these initially asymptomatic patients [1, 11]. In the retrospective analysis by Ohtsuka and Suzuki 18% of the patients experienced strangulation of the hernia content [10]. One of the patients in our study underwent surgery in the emergency setting because of incarceration of the liver due to right-sided MLH. This life-threatening complication had been rarely described in the current literature and requires immediate surgical therapy. Otherwise, the evidence for the management of MLH and especially for the surgical approach is quite low, thus official recommendations are lacking and the knowledge about appropriate treatment modalities is only based on single surgeon’s or single institutional experiences as well as case reports and small retrospective, monocentric case series without adequate long-term follow-up in the current literature (see our literature review in Table 4). According to hiatal hernia repair, trans-thoracic and trans-abdominal surgical approaches both by conventional open surgery as well as minimally-invasive surgery, have been described for MLH repair in the literature. Although in the early literature review by Horton et al., from the year 2008, a great proportion of patients with MLH were treated by conventional thoracotomy [4], patients in more recently published case reports more frequently underwent trans-abdominal, minimally-invasive approaches according to the standard surgical techniques in hiatal hernia repair [1, 5]. The latter goes in line with the recent literature overview by Ryan et al., published in 2018. Herein a growing number of 113 patients were reviewed who underwent a laparoscopic approach for MLH [11]. Hence, on comparison with trans-thoracic surgery, a trans-abdominal surgical approach for MLH provides better exposure to the hernia and better overview during hernia repair regarding relevant abdominal structures [6, 10]. Furthermore, trans-abdominal approaches allow for exclusion of hernia-associated intraabdominal pathologies or complications like strangulation and perforation [6, 10]. Besides that, some authors advocate a trans-thoracic approach by providing better overview during dissection of the hernia sac from mediastinal, pleural and pulmonary structures [4, 12]. Nevertheless, minimally-invasive trans-abdominal surgery by laparoscopy had been shown as being sufficient by providing good functional results, safe and feasible in hiatal hernia repair and, therefore, can be transferred to retrocostoxiphoid diaphragmatic hernia repair [13–15]. Furthermore, minimally-invasive thoracic and abdominal surgeries thereby provide well known advantages with regard to an ‘enhanced recovery after surgery’ when compared to conventional open approaches by thoracotomy or laparotomy [11, 16, 17]. In our study all patients were treated by trans-abdominal surgery. Our case series confirms the safety and feasibility of laparoscopy for MLH treatment in selected patients. However, patients in our study treated by open surgical trans-abdominal hernia repair more frequently received chest tubes compared to laparoscopically treated patients (*p* = 0.02). The reason was not clearly assessable retrospectively. But, perhaps the more radical resection of the hernia sac with consecutive opening of the adjacent pleura as well as the smaller expense to place any type of chest tube drainage trans-diaphragmally also in open abdominal surgery might be the possible explanations. Right-sided retrocostoxiphoid diaphragmatic hernia causing life-threatening liver incarceration in one patient of our cohort was treated by primary open trans-abdominal hernia repair with mesh augmentation from both sides of the diaphragm by laparotomy and right-sided thoracotomy. Such a ‘maximal’ surgical thoraco-abdominal approach has already been described in single case reports of congenital diaphragmatic hernia repair in adults and should be reserved for special cases [10, 18]. Nevertheless, the role of mesh reinforcement after primary suture of the hernia gaps is still disputed not only in hiatal hernia repair but also in the repair of MLH, and thus a clear evidence does not exist (see our literature review in Table 4) [1, 4, 14, 15]. Although in our case series all patients received mesh reinforcement after primary suture of the hernia, interestingly a mesh was used more frequently in minimally-invasive MLH repair by laparoscopic surgery in the retrospective studies by Horton et al. and Young et al. [1, 4]. However, it often remains unclear in most reports from the literature, if a mesh was used for augmentation after primary suture repair of the MLH or if a mesh interposition was used between the diaphragmatic gaps of the hernia (see our literature review in Table 4). The three possible surgical treatment options of extra-hiatal diaphragmatic hernia are: primary suture, primary suture with mesh reinforcement or mesh interposition to reconstruct the diaphragmatic integrity without primary closure of the hernia by sutures [11]. In their recent literature review on laparoscopic MLH repair, Ryan et al. reported a primary closure rate, a primary closure with mesh reinforcement rate and a mesh interposition rate of 34.5%, 14.1% and 49.6%, respectively [11]. In our opinion the latter is only necessary and suitable for special cases with large diaphragmatic defects, when primary reconstruction of the diaphragmatic integrity by suturing (with or without mesh reinforcement) is impractical and tension on the suture increases the risk for insufficiency. Thus some authors state that the hernial orifice above the size of 20–30 cm^2^ requires mesh interposition [19]. However, appropriate information regarding the mesh implants is frequently lacking in the literature, so it remains unclear if mesh devices implanted for MLH should have coated or uncoated layers or absorbable characteristics. Even the benefit of hernia sac resection during MLH repair in adult as well as infant patients lacks higher evidence and currently remains controversial (see our literature review in Table 4) [4, 11, 20, 21]. Radical excision and resection of the hernia sac in MLH bears the risk of violating neighboring structures like the phrenic nerve, pericardium, pleura and the lung as well [21, 22]. As this is the common practice in hiatal hernia repair at our institution we transferred it to the surgical technique of MLH repair in adult patients with good clinical results to avoid postoperative complications, derived from the hernia sac left in-situ which causes seroma, hematoma, and recurrence in the short- as well as long-term follow-up [1, 11, 21, 23]. However, most probably due to the risk of possible intraoperative complications Horton et al. have reported in their literature review the lowest rates of hernia sac resection during a laparoscopic approach (100% during a trans-thoracic approach, 82% during open abdominal and 31% during laparoscopic surgery) [4].

**Table 4:** Literature review on congenital diaphragmatic hernia in adult patients

Authors	Reference	Publication year	Study information/observational period	*n* patients	Type of hernia included	Surgical technique^a^
Young et al. [1]	Comparison of laparoscopic versus open surgical management of Morgagni hernia	2019	Retrospective, 1987–2015	43	MLH	Laparotomy: *n* = 27 Laparoscopy: *n* = 10 Thoracotomy: *n* = 6 Suture: *n* = 31 Suture + mesh: n/a Mesh: *n* = 12 Hernia sac resection: n/a
Agalar et al. [21]	Adult Morgagni hernia: a single-center experience of five cases and a review of literature	2018	Retrospective, 2009–2015	5	MLH	Laparotomy: *n* = 2 Laparoscopy: *n* = 3 Suture: *n* = 4 Suture + mesh: *n* = 0 Mesh: *n* = 1 Hernia sac resection: *n* = 0
Târcoveanuet al. [24]	Laparoscopic management in Morgagni hernia—short series and review of literature	2018	Retrospective, 2011–2017	6 of 8 patients underwent surgery	MLH	Laparotomy: *n* = 2 Laparoscopy: *n* = 4 Suture: *n* = 4 Suture + mesh: *n* = 0 Mesh: *n* = 2 Hernia sac resection: *n* = 4
Arikan et al. [5]	Morgagni’s hernia: analysis of 21 patients with our clinical experience in diagnosis and treatment	2017	Retrospective, 2003–2015	21	MLH	Laparotomy: *n* = 9 Laparoscopy: *n* = 12 Suture: *n* = 4 Suture + mesh: *n* = 6 Mesh: *n* = 11 Hernia sac resection: *n* = 17
Ohtsuka and Suzuki [10]	Right-sided Bochdalek hernia in an elderly patient: a case review of adult Bochdalek hernias from 1982 to 2015 in Japan	2017	Literature review, 1982–2015	109	Bochdalek	Trans-abdominal: *n* = 62 (by laparoscopy: *n* = 12) Trans-thoracic: *n* = 19 (by thoracoscopy: *n* = 6) Thoraco-abdominal: *n* = 16 Suture: *n* = 73 Suture + mesh: n/a Mesh: *n* = 23 Hernia sac resection: n/a
Saroj et al. [25]	Laparoscopic repair of congenital diaphragmatic hernia in adults	2016	Retrospective, 2011–2014	13^b^	Bochdalek and MLH	Laparotomy: *n* = 1 Laparoscopy: *n* = 10 Suture: *n* = 0 Suture + mesh: *n* = 10 Mesh: *n* = 3 Hernia sac resection: n/a, but usually performed
Aydin et al. [26]	Morgagni hernia: transabdominal or transthoracic approach?	2014	Retrospective, 1997–2011	20	MLH	Laparotomy: *n* = 14 Thoracotomy: *n* = 6 Suture: *n* = 19 Suture + mesh: *n* = 0 Mesh: *n* = 1 Hernia sac resection: *n* = 20
Park and Doyle [22]	Laparoscopic Morgagni hernia repair: how-I-do-it	2014	Retrospective, from 2002	13	MLH	Laparoscopy: *n* = 13 Suture: *n* = 13 Suture + mesh: *n* = 0 Mesh: *n* = 0 Hernia sac resection: n/a, but is recommended
Abraham et al. [6]	Morgagni−Larrey hernia—a review of 20 cases	2012	Retrospective, 1997–2010	20^c^	MLH	Trans-abdominal surgery Laparotomy: n/a Laparoscopy: n/a Suture: *n* = 10 Suture + mesh: *n* = 4 Mesh: *n* = 6 Hernia sac resection: n/a
Aghajanzadeh et al. [20]	Clinical presentation and operative repair of Morgagni hernia	2012	Retrospective, 1996–2010	36	MLH	Laparotomy: *n* = 26 Thoracotomy: *n* = 6 Thoraco-abdominal: *n* = 4 Suture: *n* = 36 Suture + mesh: *n* = 0 Mesh: *n* = 0 Hernia sac resection: *n* = 0
Gedik et al. [27]	A review of Morgagni and Bochdalek hernias in adults	2011	Retrospective, 2000–2010	20	Bochdalek and MLH	Laparotomy: *n* = 19 Thoraco-abdominal: *n* = 1 Suture: *n* = 13 Suture + mesh: *n* = 1 Mesh: *n* = 6 Hernia sac resection: n/a
Altinkaya et al. [28]	Morgagni hernia: diagnosis with multidetector computed tomography and treatment	2009	Retrospective, 2004–2009	6 of 12 patients underwent surgery	MLH	Laparotomy: *n* = 3 Laparoscopy: *n* = 2 Thoracotomy: *n* = 1 Suture: *n* = 5 Suture + mesh: *n* = 0 Mesh: *n* = 1 Hernia sac resection: *n* = 0
Palanivelu et al. [29]	Laparoscopic repair of adult diaphragmatic hernias and eventration with primary sutured closure and prosthetic reinforcement: a retrospective study	2009	Retrospective, 1995–2007	21^d^	Bochdalek and MLH	Laparoscopy: *n* = 21 Suture: *n* = 3 Suture + mesh: *n* = 18 Mesh: *n* = 0 Hernia sac resection: *n* = 4
Horton et al. [4]	Presentation and management of Morgagni hernias in adults: a review of 298 cases	2007	Literature review, 1951–2006	298	MLH	Laparotomy: *n* = 30% Laparoscopy: *n* = 17% Thoracotomy: *n* = 49% Thoracoscopy: *n* = 0.7% Suture: *n* = 29% Suture + mesh: *n* = 7% Mesh: *n* = 64% Hernia sac resection: 75.2%
Yilmaz et al. [30]	Transabdominal approach in the surgical management of Morgagni Hernia	2007	Retrospective, 1999–2005	11	MLH	Laparotomy: *n* = 11 Suture: *n* = 10 Suture + mesh: *n* = 0 Mesh: *n* = 1 Hernia sac resection: n/a
Durak et al. [31]	Laparoscopic repair of Morgagni hernia	2007	Retrospective, 2001–2005	5	MLH	Laparoscopy: *n* = 5 Suture: *n* = 1 Suture + mesh: *n* = 0 Mesh: *n* = 4 Hernia sac resection: *n* = 0
Ambrogi et al. [12]	Transthoracic repair of Morgagni’s hernia: a 20-year experience from open to video-assisted approach	2007	Retrospective, 1985–2005	22	MLH	Thoracotomy: *n* = 17 Transxiphoid hand-assisted thoracoscopy: *n* = 5 Suture: *n* = 16 Suture + mesh: *n* = 4 Mesh: *n* = 2 Hernia sac resection: n/a
Yavuz et al. [32]	Laparoscopic repair of Morgagni hernia	2006	Retrospective, 2002–2004	5	MLH	Laparoscopy: *n* = 5 Suture: *n* = 0 Suture + mesh: *n* = 0 Mesh: *n* = 5 Hernia sac resection: *n* = 0

*MLH* Morgagni−Larrey hernia; *n/a* Not assessable.

^a^Most common type of surgery and most common technique of hernia repair (primary suture ± mesh reinforcement or mesh interposition)

^b^Congenital derived diaphragmatic hernia in only five cases

^c^Post-traumatic in five patients

^d^Post-traumatic in three patients

Pulmonary complications and pleural effusions, which require thoracic drainage postoperatively, are the most common clinical problems after MLH repair [4]. As reported in the current literature as well as in the present study, recurrence rates after MLH repair are rather low, but it has to be taken into consideration that the evidence is only based on retrospectively conducted case series, in which long-term follow-up might be insufficient. A further limitation in studies of the current literature as well as in our patient cohort regarding long-term follow-up is that it is mainly based on clinical examination of the patients, who are asymptomatic in almost one third of the cases.

In conclusion the evidence of MLH repair in adult patients is low. The limitations of the reported case series in the recent literature regarding MLH repair in adults, including the present study, are small sample sizes, lack of quality of life appraisal and long-term follow-up of the patients, the retrospective nature of the studies as well as the long observational period with different standards in the surgical therapy of diaphragmatic hernia repair over time. However, case series in the recent literature as well as the present study show the safety and feasibility of trans-abdominal surgical approaches for the treatment of MLH in selected adult patients. By analogy with current treatment standards of hiatal hernia repair, MLH in adult patients should be repaired whenever possible by minimally-invasive surgery by experienced surgeons. Therefore, the role and evidence of mesh augmentation after primary suture repair as well as the benefit of hernia sac excision are still disputed and should be further evaluated.
